# Reversible Human Immunodeficiency Virus Type-1 Latency in Primary Human Monocyte-Derived Macrophages Induced by Sustained M1 Polarization

**DOI:** 10.1038/s41598-018-32451-w

**Published:** 2018-09-24

**Authors:** Francesca Graziano, Giulia Aimola, Greta Forlani, Filippo Turrini, Roberto S. Accolla, Elisa Vicenzi, Guido Poli

**Affiliations:** 1 Division of Immunology, Transplantation and Infectious Diseases, San Raffaele Scientific Institute, Milano, Italy; 2 Department of Medicine and Surgery, University of Insubria, Varese, Italy; 3 Vita-Salute San Raffaele University School of Medicine, Milano, Italy; 4 Present Address: Institute Curie Laboratoire Immunité et Cancer – INSERM U932, 26 rue d’Ulm, 75248 Paris cedex 05, Paris, France

## Abstract

We have reported that short-term stimulation of primary human monocyte-derived macrophages (MDM) with interferon-γ (IFN-γ) and tumor necrosis factor-α (TNF-α), i.e. M1 polarization, leads to a significant containment of virus replication. Here we show that M1-MDM restimulation with these cytokines 7 days after infection (M1^2^ MDM) promoted an increased restriction of HIV-1 replication characterized by very low levels of virus production near to undetectable levels. In comparison to control and M1-MDM that were not restimulated, M1^2^ MDM showed a stronger reduction of both total and integrated HIV DNA as well as of viral mRNA expression. M1^2^ MDM were characterized by an upregulated expression of restriction factors acting at the level of reverse transcription (RT), including apolipoprotein B mRNA editing enzyme, catalytic polypeptide-like 3A (APOBEC3A) and APOBEC3G, but not SAM domain and HD domain-containing protein 1 (SAMHD1). M1^2^ MDM also showed an increased expression of Class II Transactivator (CIITA) and Tripartite Motif22 (TRIM22), two negative regulators of proviral transcription, whereas expression and phosphorylation of transcriptional inducers of HIV-1, such as nuclear factor kB (NF-kB) and signal transducer and activator of transcription 1 (STAT1), were not impaired in these cells. The almost quiescent state of the infection in M1^2^ MDM was promptly reversed by coculture with mitogen-stimulated leukocytes or cell incubation with their filtered culture supernatant. M1^2^ MDM harbored replication-competent HIV-1 as virus spreading following cell stimulation was fully prevented by the RT inhibitor lamivudine/3TC. Selective reactivation of proviral expression in M1^2^ MDM, but not in control or in M1-MDM that were not restimulated, was confirmed in cells infected with single round Vesicular Stomatitis Virus-G-pseudotyped HIV-1. Thus, M1^2^ MDM represent an *in vitro* model of reversible, almost quiescent HIV-1 infection of primary human macrophages that could be further exploited for “Cure” related investigations.

## Introduction

The human immunodeficiency virus type-1 (HIV-1) infects cells expressing the primary entry receptor CD4 and either CCR5 or CXCR4 as mandatory entry coreceptors. This entry requirements restrict the number of cells susceptible to HIV-1 infection mostly to a major subset of T lymphocytes and to myeloid cells, with the exception of the astrocytes of the central nervous system (CNS) that are infected *in vivo* and infectable *in vitro* via a CD4/chemokine receptor independent route^1^.

In addition to entry requirements, infection of T cells and myeloid cells share several features, such as the capacity to counteract active virus replication by means of constitutively expressed restriction factors, including members of the apolipoprotein B mRNA editing enzyme, catalytic polypeptide-like (APOBEC) family, usually promoting G-to-A hypermutations in the viral sequence during reverse transcription process^2,3^, or SAM domain and HD domain-containing protein 1 (SAMHD1), a phosphohydrolase that curtails the reverse transcription step in the virus life cycle by depleting the pool of dideoxynucleoeside triphosphates^4^. Once integrated as a provirus, HIV-1 can undergo active transcription leading to translation and assembly of its viral proteins with genomic RNA at the plasma membrane in order to release new progeny virions or it can be silenced by either epigenetic mechanisms, lack of positive transcription factors and/or dominance of negative transcription factors^5^. In this regard, a crucial role is played by the provirus-encoded protein Tat that acts as anti-terminator of viral transcripts by promoting the assembly of the Positive Transcriptional Elongation Factor b (P-TEFb) complex, composed of cyclin T1 and cyclin-dependent kinase 9 (CDK9)^5,6^.

HIV-1 replication *in vivo* leads to a progressive and selective depletion of CD4^+^ T lymphocytes leading to the acquired immunodeficiency syndrome (AIDS) in the absence of combination antiretroviral therapy (cART). Although the precise mechanism(s) of CD4^+^ T cell depletion *in vivo* are still debated^7^, *in vitro* infection of activated primary CD4^+^ T cells is associated with virus-induced cytopathicity and cell death. In contrast, no depletion of circulating monocytes or tissue-associated macrophages is usually seen in infected individuals whereas *in vitro* infection of primary human monocyte-derived macrophages (MDM) does not lead to a significant cytopathicity and cell death^8,9^. Furthermore, both *in vivo* and *in vitro* macrophage infections are characterized by the active accumulation of mature virions in subcellular compartments generated as invaginations of the plasma membrane nowadays referred to as virus-containing compartments (VCC)^9,10^, a feature not observed in CD4^+^ T cells. These distinctive features of macrophage infection render them potential candidates for contributing to viral persistence in individuals profoundly depleted of CD4^+^ T cells as well as in those receiving cART^1^.

The potential role of infected macrophages as long-lived reservoirs of silent infectious provirus is challenged by several observations, including the uncertainty of the lifespan of infected tissue-resident macrophages, potential “false positive” findings in tissue preparations due to the presence of residual infected CD4^+^ T cells^11^, phagocytosis of infected CD4^+^ T cells (with unclear consequences in terms of macrophage infection or virus elimination)^12,13^, and the lack of a molecular signature that would unequivocally demonstrate the non-CD4^+^ T cell origin of virus rebounding after cART suspension, as reviewed^9,14–16^. In support of a relevant role of infected macrophages in HIV-1 persistency there are different observations in macaques experimentally infected with the simian immunodeficiency virus (SIV)^17,18^, a close relative of HIV-1, as well as clinical evidence of active HIV-1 replication and/or HIV-related inflammation in the CNS occurring in a fraction of individuals with suppressed viremia by cART^19^. The hypothesis that, in addition to CD4^+^ T cells, macrophages could be prominent contributors to the cART-resistant reservoir of infectious proviruses has been further supported by the recent evidence that mice with severe combined immunodeficiency (SCID) reconstituted with human myeloid cells in the absence of CD4^+^ T cells can undergo both acute and chronic HIV-1 infection with establishment of an inducible HIV-1 reservoir, at least in some animals^20^.

An additional element fostering the perplexity on the role of tissue-associated macrophages in persistent HIV-1 infection is the lack of robust *in vitro* models based on primary cells rather than on immortalized myeloid cell lines. In this regard, we have previously shown that stimulation of either primary human MDM^21^ or monocytes^10^ with potent pro-inflammatory cytokines, namely interferon-γ (IFN-γ) and tumor necrosis factor-α (TNF-α), before infection leads to a significant containment of virus replication. This HIV-restrictive profile was characterized by several features, including downregulation of the CD4 molecule from the cell surface, upregulated secretion of CCR5-binding chemokines and induction of the expression of the putative restriction factor APOBEC3A^22^. In the present study, we have investigated the potential consequences of restimulating infected M1-MDM with IFN-γ and TNF-α, as these cytokines are credited with latency-reversal capacity driven by activation of STAT1 and NF-kB transcription factors, respectively^23–26^. Quite surprisingly, we observed that a second stimulation of infected M1-MDM with these pro-inflammatory cytokines several days after infection, a condition here defined as “M1 squared (M1^2^) MDM”, further inhibited HIV-1 replication down to very low levels for several weeks. However, this quasi-silent infection was fully reversed leading to rapid viral spreading by either cocultivation with activated peripheral blood mononuclear cell (PBMC) or by incubation with their culture supernatants, an effect that was prevented by the reverse transcriptase (RT) inhibitor lamivudine/3TC. As both the selective repression of virus expression and its reactivation in M1^2^ MDM upon PHA blast coculture were reproduced in cells infected with single round Vesicular Stomatitis Virus-G (VSV-G) pseudotyped HIV-1, M1^2^ MDM represent an *in vitro* model that could be further exploited to decipher the molecular and immuno-pharmacological features of reversible latency/low levels HIV-1 production in primary human macrophages.

## Results

### Restimulation of infected M1-MDM with pro-inflammatory cytokines leads to a stronger containment of HIV-1 replication

**Figure 1:**
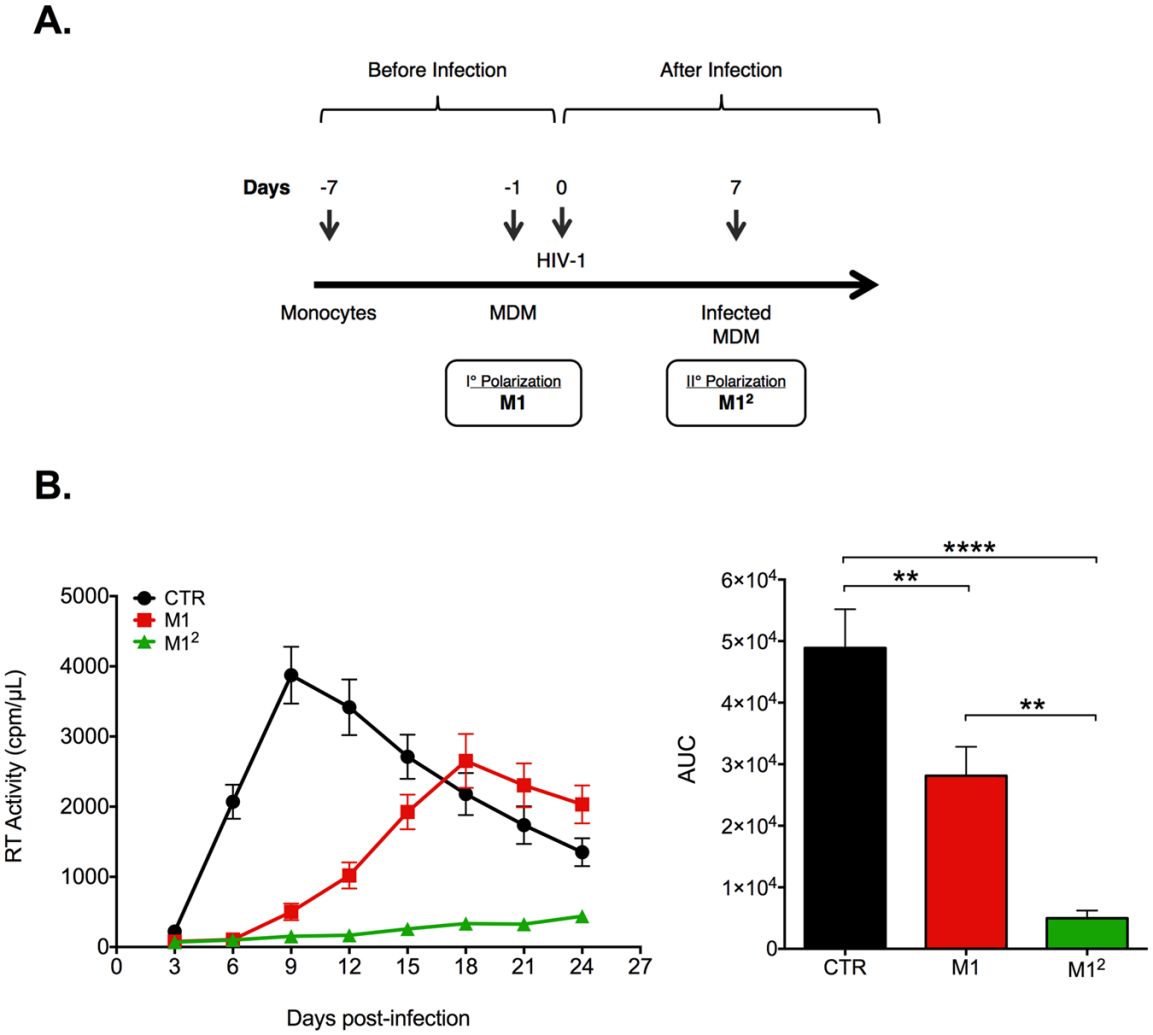
M1-MDM stimulation with polarizing cytokines after infection further inhibits HIV-1 replication. (**A**) Experimental scheme. Seven days-old MDM were either left unstimulated (CTR) or were stimulated with IFN-γ plus TNF-α (M1-MDM) 18 h before HIV-1 infection (m.o.i = 0.1). A second stimulation of M1-MDM with the same polarizing cytokines was performed 7 days after infection (M1^2^ MDM); as for the first stimulation, cytokine-enriched medium was removed 18 h later and replaced with complete medium. (**B**) M1^2^ MDM show a superior capacity of curtailing HIV-1 replication than M1-MDM. Left panel: kinetics of supernatant-associated RT activity; right panel: Area under curve (AUC); statistical analysis by One-way ANOVA; **p < 0.01, ****p < 0.0001 (n = 8).

**Figure 2:**
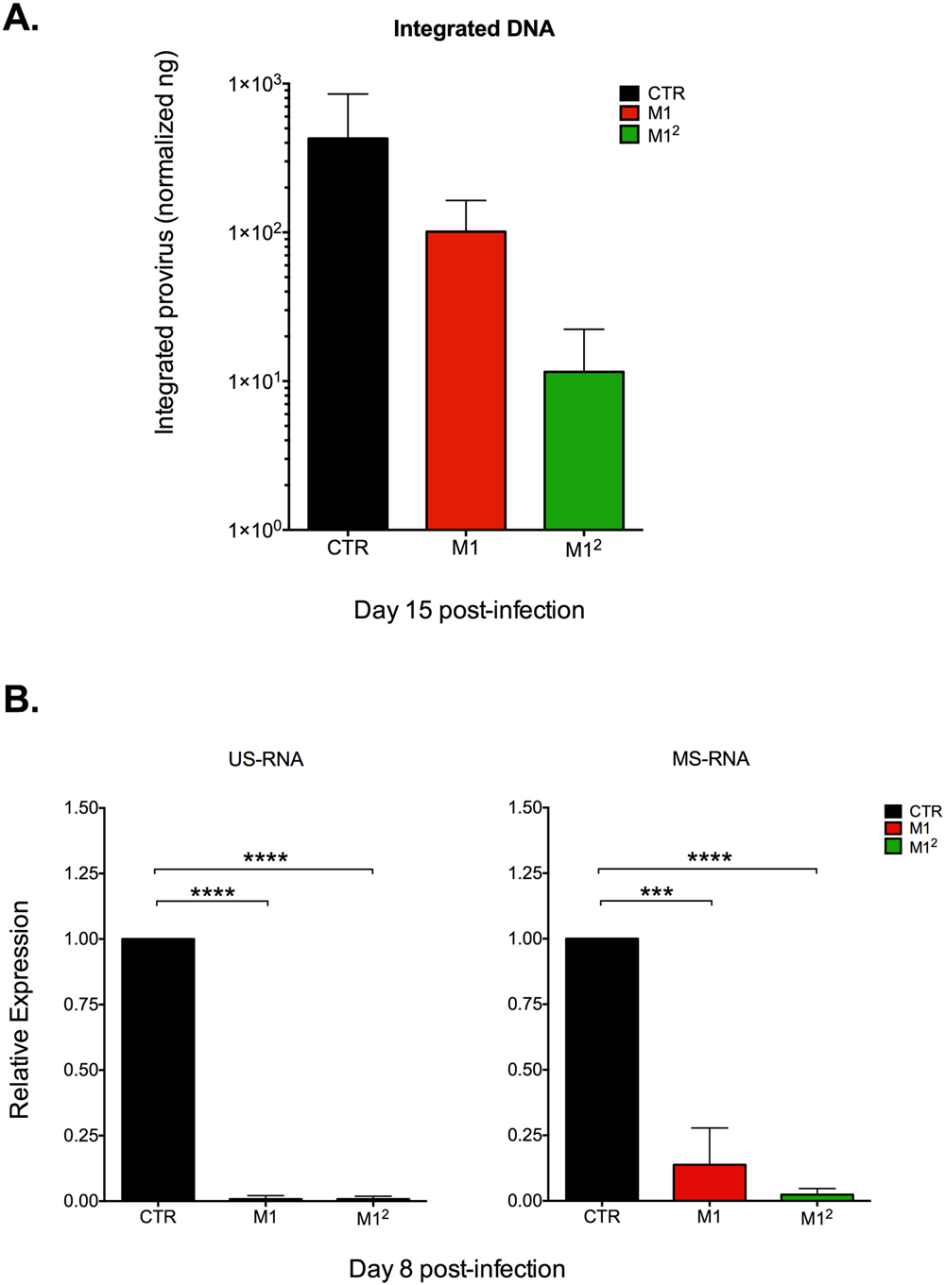
M1^2^ MDM show reduced levels of integrated (proviral) HIV DNA than CTR and M1-MDM together with a silent profile of proviral transcription. (**A**) Alu-PCR was evaluated by semi-quantitative determination of HIV integrated provirus normalized to mitochondrial DNA (ng/ng) in extracts from MDM obtained 15 days after infection. Reduced levels of integrated provirus were observed in M1-MDM and, particularly, in M1^2^ MDM that, however, were not statistically significant by paired ANOVA; the results show the means ± SD of infections of cells obtained from 5 independent donors. (**B**) Silent transcriptional profile of M1^2^ MDM after cytokine restimulation. Cytokine restimulation of M1-MDM 8 days after infection did not result in increased levels of either US or MS HIV-1 RNA. Bar graphs represent the fold difference of M1 and M1^2^ macrophages in respect with unpolarized CTR cells Days post-infection: 8 (n = 2), 12 (n = 8), 18 and 21 (n = 4); statistical analysis by One-way ANOVA *p < 0.05, **p < 0.01; ***p < 0.001.

Having described that M1 polarization of primary human MDM leads to a reduction of HIV-1 replicative capacity^21,22^, we have investigated the consequences of reexposing M1-MDM that were infected 7 days earlier by the R5 HIV-1_Bal_ strain to the same cytokines (Fig. 1A), an experimental condition further referred to as “M1^2^ MDM”. We confirmed that M1 stimulation before infection lead to a significant reduction of virus replication *vs*. unstimulated, control (CTR) MDM (Fig. 1B). However, a much greater inhibition of virus propagation, measured both in terms of kinetics and area under the curve (AUC), was consistently observed in M1^2^ MDM *vs*. M1-MDM obtained from multiple seronegative donors (Fig. 1B). The strong inhibitory profile associated with M1^2^ MDM was not caused by cytotoxic effects of cytokine stimulation. Freeze-fracture experiments were performed in order to quantify the levels of cell-associated *vs*. released virions, as reported^27^, in these experimental conditions. However, no detectable cell-associated RT activity was observed in both M1-MDM and M1^2^ MDM (Fig. 1S). Furthermore, if infected CTR MDM were stimulated 7 days post-infection with M1 cytokines only a moderate inhibition of virus replication was observed (Fig. 2S).

Thus, a second stimulation of infected M1-MDM with the same pro-inflammatory cytokines several days after infection further strengthened their capacity to contain virus replication.

### M1^2^ MDM are characterized by persistently low levels of total and integrated HIV-1 DNA together with a silent transcriptional profile

**Figure 3:**
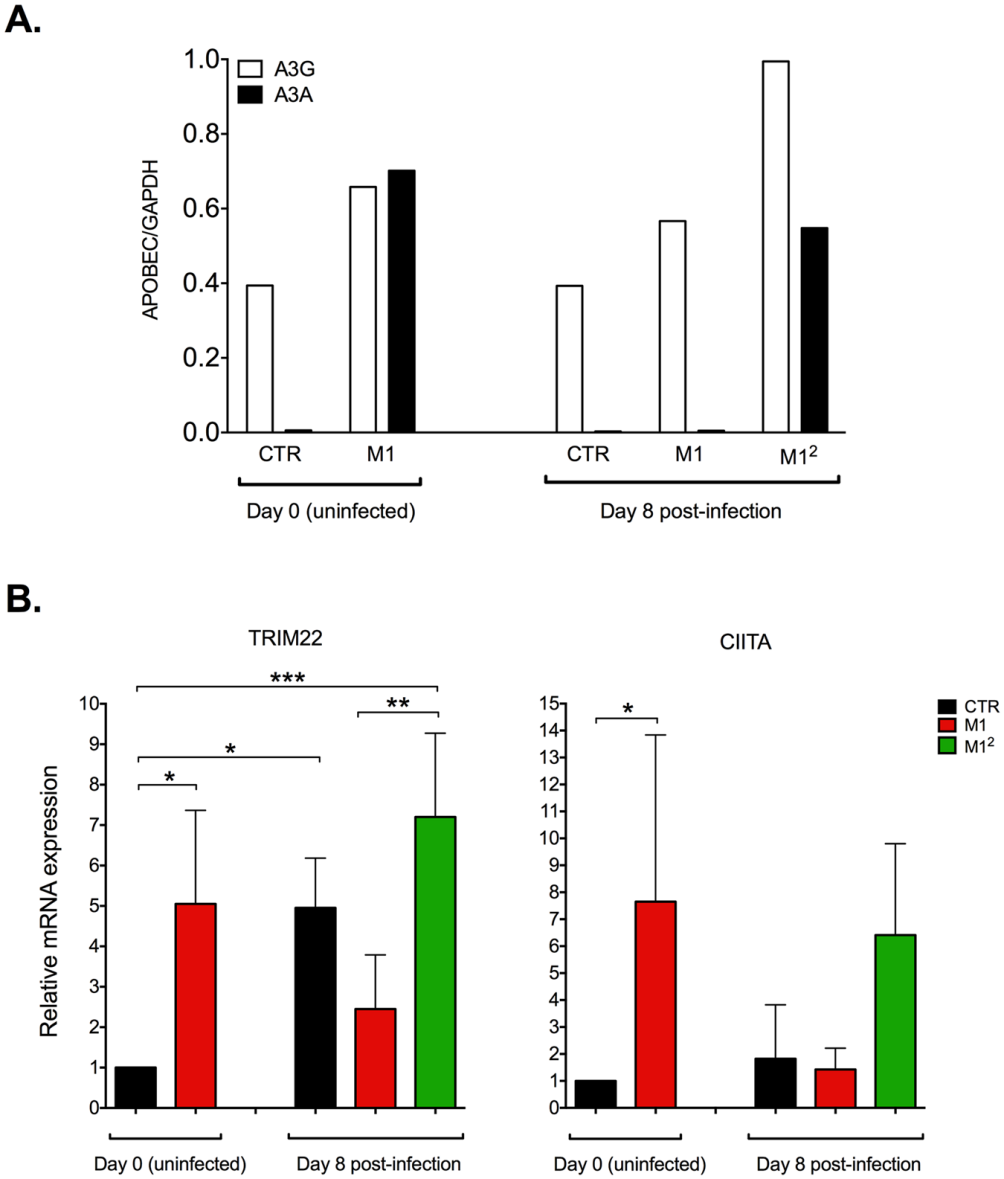
Upregulation of HIV-1 restriction factors in M1^2^ MDM. (**A**) Expression of APOBEC3A (A3A) and APOBEC3G (A3G). Cytokine stimulation induced the expression of A3A and upregulated that of A3G both before infection and 8 days after. The quantification of the original Western blot shown as Fig. 5S was obtained with the use of the ImageJ software (version 1.47 v, WS Rasband ImageJ, NIH; http://rsb.info.nih.gov). (**B**) Upregulation of TRIM22 and CIITA mRNA expression. Cytokine stimulation induced the expression of both negative regulators of proviral transcription before and 8 days after infection; the levels of mRNA expression of TRIM22, but not those of CIITA, were also increased in CTR MDM 7 days after infection. Bar graphs represent the fold difference as compared to CTR cells at day 0 prior to infection. Statistical analysis by One-way ANOVA; *p < 0.05, **p < 0.01; ***p < 0.001 (n = 4).

The profound containment of HIV-1 replication in M1^2^ MDM observed by quantification of RT activity was consistent with a significant reduction of the levels of total HIV-1 DNA in comparison to both CTR, infected MDM and also to M1-MDM that were not restimulated (Fig. 3S). M1-MDM showed an initial containment of viral DNA accumulation, as reported^22^; however, as early as 12 days after infection, their HIV DNA levels became superimposable to those of CTR MDM. In contrast, M1^2^ MDM maintained significantly lower levels of HIV-1 DNA (although with some fluctuations) at least up to 21 days after infection (Fig. 3S). Reduced levels of proviral DNA (however not reaching statistical significance) were detected by Alu-PCR in M1^2^ MDM *vs*. CTR and M1-MDM 15 days post-infection (i.e. 8 days after cytokine restimulation in the case of M1^2^ MDM; Fig. 2A).

**Figure 4:**
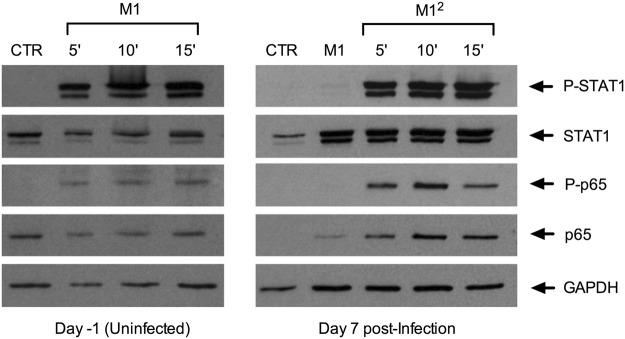
Activation of STAT1 and NF-kB in M1 and M1^2^ MDM. Cytokine stimulation induced activation of STAT-1 and NF-kB in both uninfected (left panel) and 7-days infected (right panel) MDM. Increased expression of STAT-1 was also observed in M1-MDM 7 days after infection (right panel). These results were obtained with the cells of a single donor representative of 4 independently tested.

When the levels of both unspliced (US) and multiply-spliced (MS) viral RNAs were quantified in the different experimental conditions 8 days post-infection (i.e. one day after restimulation with pro-inflammatory cytokines in the case of M1^2^ MDM) both M1 and M1^2^ MDM showed significantly lower levels of viral transcripts in comparison to those of CTR infected MDM (Fig. 2B). This quasi-silent transcriptional profile of M1^2^ MDM was confirmed also at later time points both in the presence or absence of the RT inhibitor lamivudine/3TC (Fig. 4S).

Altogether, these results support the hypothesis that M1^2^ MDM may represent an *in vitro* model of latent HIV-1 infection of primary human macrophages.

### Upregulation of HIV-1 restriction factors in M1^2^ MDM

**Figure 5:**
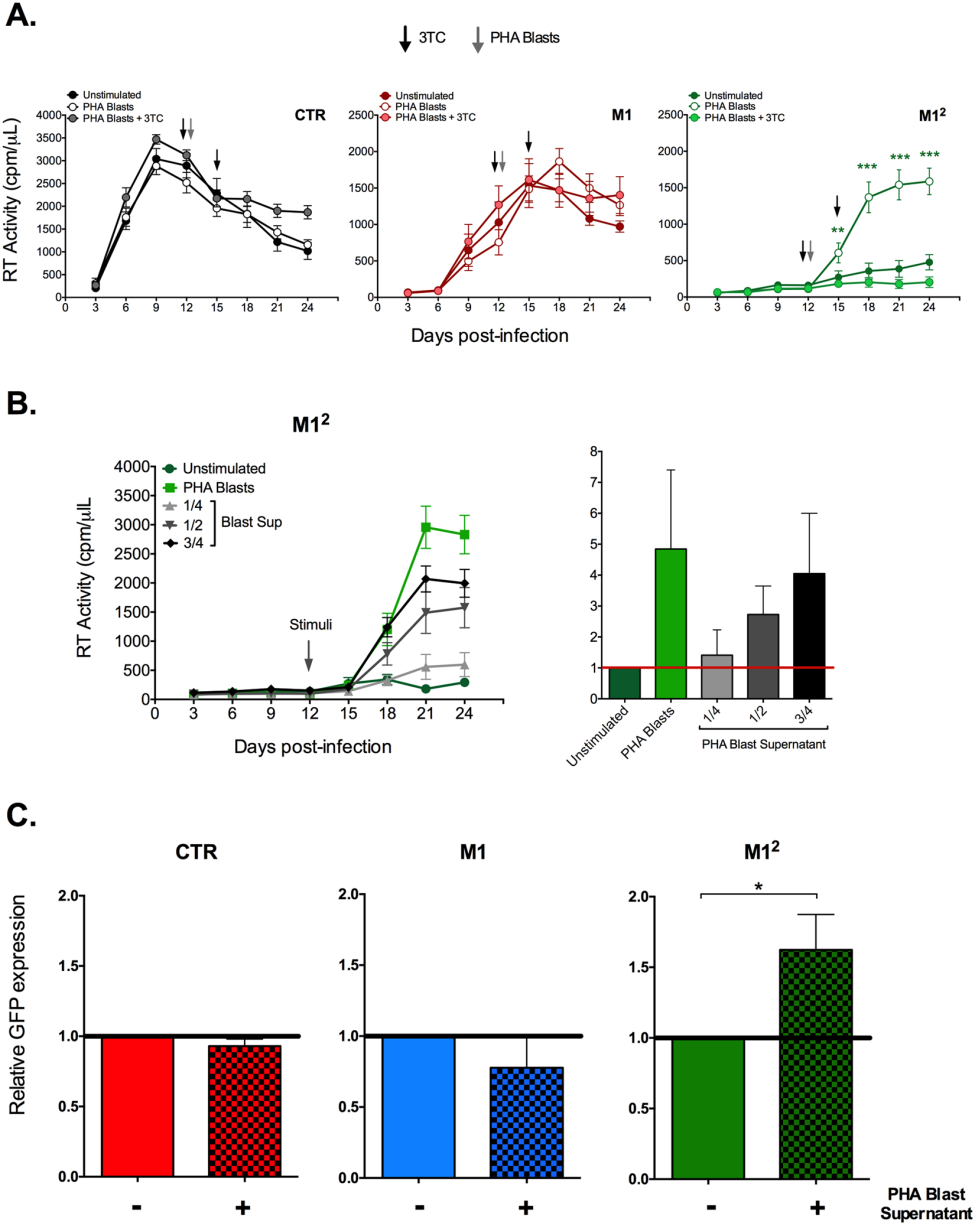
Selective induction of HIV-1 replication (or expression of VSV-G pseudotyped virus) in M1^2^ MDM stimulated by allogeneic PHA blasts or their supernatant and its selective prevention by lamivudine/3TC. (**A**) Kinetics of RT activity. PHA blasts upregulated virus replication in M1^2^ MDM, but not in CTR or M1-MDM; the arrowheads indicate the timing of PHA blast addition to the infected MDM cultures. Mean ± SEM of 7 independent donors is reported. Comparison of different time points between M1 *vs*. M1^2^ plus PHA blasts was performed using One-way ANOVA. ***p < 0.001. The addition of lamivudine/3TC (1 µM - black arrowheads) did not substantially affect virus production in CTR and M1-MDM, but fully prevented HIV-1 replication in M1^2^ MDM. (**B**) Left panel: kinetics of RT activity; right panel: RT activity ratios relative to unstimulated M1^2^ MDM calculated at peak virus replication in the different conditions (n = 2). The arrowhead indicates the timing of stimuli addition to the infected MDM cultures. (**C**) CTR, M1-MDM and M1^2^ MDM were infected with a VSV-G pseudotyped virus expressing eGFP. Thirteen days after infection the cells were incubated with Blast Sup for 72 h before determining the levels of eGFP expression. Both M1-MDM and M1^2^ MDM showed reduced levels of GFP expression vs. CTR cells, but only M1^2^ MDM were significantly enhanced in terms of virus expression by incubation with Blast Sup (p < 0,05 by T-test, n = 3; see also Fig. 9S).

We next investigated whether the strong containment of virus replication observed in M1^2^ MDM was associated with an increased expression of restriction factors known to counteract HIV-1 infection by acting at the level of reverse transcription, before proviral integration, namely APOBEC3A, APOBEC3G and SAMHD1^28^. As we^22^ and others^29^ have originally reported, APOBEC3A was not expressed in uninfected control MDM whereas APOBEC3G was visible by Western blotting as a faint band (Fig. 5S). M1 polarization induced a modest upregulation of APOBEC3G, but a clear-cut expression of APOBEC3A (Figs 3A and 5S), as published^22^. Eight days after infection, the levels of expression of these two restriction factors in M1-MDM returned to levels comparable to those observed at baseline; conversely, M1^2^ MDM showed significantly higher levels of both restriction factors than both CTR and M1-MDM (Figs 3A and 5S). In contrast, SAMHD1 was clearly expressed by MDM, but it was not significantly modulated in these experimental conditions (Fig. 6S).

It is well known that macrophage stimulation with IFN-γ and TNF-α triggers multiple signaling pathways resulting in cell activation according to a pro-inflammatory mode^30^. Of interest, some factors either induced or post-translationally modified by M1 polarization have been previously reported to modulate HIV-1 proviral transcription either in a positive or negative fashion. In particular, IFN-γ was reported to upregulate the expression of TRIM22 and CIITA, two inducible restriction factors acting as repressors of proviral transcription by independent mechanisms^31–34^. The expression of both TRIM22 and CIITA mRNAs was indeed promptly upregulated after 18 h of cell stimulation of MDM with M1 cytokines before infection (Fig. 3B). Of interest, CTR MDM showed a significant increase in TRIM22 mRNA expression in comparison to their basal levels 8 days after infection. Infected M1^2^ MDM expressed significantly higher levels of TRIM22 mRNA *vs*. M1-MDM that were not restimulated (Fig. 3B, left panel). CIITA mRNA expression showed a partially different pattern in that, like TRIM22, it was strongly upregulated in uninfected MDM by stimulation with M1 cytokines; however, unlike TRIM22, its levels returned to baseline in both CTR and M1-MDM 8 days after infection (Fig. 3B, right panel) whereas they were strongly induced by cytokine restimulation in M1^2^ MDM (Fig. 3B, right panel). In contrast, no significant changes in the low levels of either cyclin T1 or CDK9 (forming the P-TEFb complex) were observed in these different experimental conditions (Fig. 7S).

Thus, the quasi-silent profile of HIV expression in M1^2^ MDM was associated with an increased expression of APOBEC3A and APOBEC3G as well as of TRIM22 and CIITA, two IFN-inducible factors exerting a negative effect on proviral transcription.

### The lack of HIV-1 expression in M1^2^ MDM is not explained by an impaired activation of STAT1 and NF-kB

Both IFN-γ and TNF-α have been earlier described as potent inducers of HIV-1 transcription and expression acting via activation of STAT1 and of the canonical NF-kB pathway, respectively, in different cell models, including interleukin-2 (IL-2) activated PBMC^35^ and myeloid cells^23–26^. Therefore, we have investigated whether the silent transcriptional and expression profiles observed in M1^2^ MDM could be accounted for by an impaired activation of these two transcription factors.

When uninfected MDM were stimulated with IFN-γ and TNF-α, a prompt activation of STAT1, measured in terms of phosphorylation of this transcription factor, was observed 5 min after cytokine stimulation and remained clearly visible after 10 additional min (Fig. 4, left panel). This finding was consistent with the observation that the upregulation of CIITA expression upon cell stimulation with IFN-γ requires STAT1 activation^36^. Furthermore, the phosphorylated form of NF-kB p65 became promptly visible upon TNF-α stimulation of these cells. After infection and 7 additional days of culture, no evidence of residual activation of either STAT1 or NF-kB was observed in both control and M1-MDM. However, restimulation of these latter cells with IFN-γ and TNF-α resulted in the rapid and robust activation of both STAT1 and NF-kB (Fig. 4, right panel). As previously reported^37^, an increased expression of STAT1 was observed in M1-MDM 7 days after infection (Fig. 4, right panel).

Thus, the silent HIV-1 transcriptional profile observed in M1^2^ MDM was not consequent to absent or impaired activation of STAT1 and NF-kB, two transcription factors endowed with the capacity of triggering or potentiating proviral transcription.

### Allogeneic mitogen-stimulated PBMC or their culture supernatant reactivate HIV-1 replication in M1^2^ MDM, but not in CTR or M1-MDM

In order to verify whether the almost silent HIV-1 replicative profile of infected M1^2^ MDM could be reversed by cell stimulation, we incubated both CTR and polarized infected MDM with allogeneic PBMC that were activated 72 h before with the mitogen phytohemagglutinin (PHA blasts). Of note, PHA blasts were not resuspended in medium containing IL-2 in order to curtail cell proliferation and viral spreading in these cells. In this regard, we observed that IL-2-stimulated PHA blasts rapidly overcame adherent M1^2^ MDM leading to their elimination likely by cell fusion, release of cytotoxic cytokines, or activation-induced cell death (data not shown).

Incubation of infected CTR or M1-MDM with PHA blasts did not modify their levels of RT activity production (Fig. 5A); a similar observation was made by analyzing the levels of HIV DNA in the same experimental conditions (Fig. 8S). In sharp contrast, incubation of infected M1^2^ MDM with PHA blasts resulted in a prompt and robust release of RT activity in culture supernatants in association with a clear-cut rise in the levels of cell-associated HIV DNA (Figs 5A and 8S, respectively).

In order to determine whether the released virus was produced from persistently infected MDM or by propagation of a replication-competent HIV-1, these experiments were also conducted in the presence of the RT inhibitor 3TC/lamivudine (1 µM). Of interest, a clear-cut dichotomous profile was observed in that the antiviral agent did not significantly affect virus production in unstimulated or PHA blast-stimulated CTR and M1-MDM, but abrogated virus replication in M1^2^ MDM both in unstimulated and stimulated conditions (Fig. 5A).

Next, we investigated whether cell-to-cell contact with PHA blasts was required for virus reactivation in infected M1^2^ MDM. To this purpose, M1^2^ MDM cells were incubated with different dilutions of filtered PHA blast supernatants (Blast Sup). Blast Sup upregulated virus production from infected M1^2^ MDM in a dilution-dependent manner, although, as expected, with a lower potency than that observed with PHA blast coculture with M1^2^ MDM (Fig. 5B).

### PHA blast supernatant selectively reactivates VSV-G pseudotyped HIV-1 expression in M1^2^ MDM, but not in CTR or M1-MDM

In order to consolidate the profile observed with replication-competent HIV-1 in terms of restricted expression and proviral reactivation by cell stimulation with allogeneic PHA blasts culture supernatants, these experiments were also conducted after infection of CTR, M1 and M1^2^ MDM with VSV-G pseudotyped virus expressing enhanced Green Fluorescent Protein (eGFP) that bypasses the CD4 and CCR5 requirement for viral entry and establishes a highly efficient single round infection of these cells.

As reported^22^, the M1-dependent restriction was observed also using this experimental approach; indeed, almost 50% of the CTR MDM expressed eGFP 13 days post-infection without any influence of 72 h incubation with Blast Sup (Fig. 9S). A similar observation was made for M1-MDM that were not restimulated and whose lower levels of eGFP expression *vs*. those of CTR cells were not enhanced by Blast Sup (Figs 5C and 9S), thus recapitulating what observed with replication-competent HIV-1 (Fig. 5A). In contrast, consistently with the results obtained with replication-competent HIV-1 (Fig. 5A), M1^2^ MDM showed a moderate, but statistically significant, enhancement of virus expression upon Blast Sup stimulation (Figs 5C and 9S).

These observations together provide substantial evidence that HIV-1 infection of M1^2^ MDM established a state of persistent low levels HIV-1 replication likely including cell harboring latent, replication-competent proviruses that could be reversed by immunologic stimulation to produce infectious virus.

## Discussion

In the present study, we have further investigated our previous observation that short-term stimulation of human MDM with pro-inflammatory cytokines (IFN-γ and TNF-α), i.e. M1 polarization, before infection induces a partial restriction of HIV-1 replication. By restimulating M1-MDM with the same cytokines 7 days after infection (M1^2^ MDM), we have consistently observed a more profound restriction of virus replication approaching the limits of RT activity detection in culture supernatants. Together with a silent profile of proviral transcription, M1^2^ MDM were characterized by lower levels of total and integrated HIV DNA in comparison to CTR infected MDM that did not undergo M1 polarization and also of M1-MDM that were not restimulated with the pro-inflammatory cytokines after infection. Infected M1^2^ MDM showed an upregulation of several well-known intracellular inhibitors of virus replication, acting either before (APOBEC3A, APOBEC3G) or after (TRIM22 and CIITA) proviral integration, although the activation of both of STAT1 and canonical NF-kB, known inducers of proviral transcription, was not impaired in these cells. Activation of HIV-1 expression was promptly triggered in M1^2^ MDM, but not in control or M1-MDM, by either cocultivation with allogeneic PHA blasts or incubation with their culture supernatants (Blast Sup). This effect was fully prevented in M1^2^ MDM, but not in CTR or M1-MDM, by the RT inhibitor lamivudine/3TC therefore indicating that they harbored replication-competent virus. Proviral reactivation by Blast Sup was also selectively observed in M1^2^ MDM, but not in CTR or M1-MDM, infected with single-round VSV-G pseudotyped HIV-1.

The observation that cART suspension in individuals with fully controlled levels of HIV-1 viremia almost invariably results in the rapid resurgence of virus replication and disease progression demonstrates a central role of viral reservoirs insensitive to antiretroviral agents in life-long persistency of HIV-1 infection^38^. The demonstration of the existence of a small pool of long-lived, resting memory CD4^+^ T lymphocytes harboring replication-competent provirus has provided the first demonstration of a viral reservoir^39–41^. Several contributions have refined the composition of latently infected CD4^+^ T cell subsets highlighting a prominent role of follicular T helper cells^42^, and a more accurate estimate of the size of the replication-competent proviral reservoir^43,44^. In contrast, the contribution of myeloid cells, and of tissue macrophages in particular, has remained elusive with the partial exception of the central role of microglial cells, together with astrocytes, in the pathogenesis of brain infection with compelling evidence obtained in SIV-infected macaques^17,18^ and in individuals with viremia suppressed by cART, showing evidence of either active virus replication or HIV-related inflammation in the CNS^19^.

A component of the perplexity on the potential role of macrophages in HIV-1 persistency is the lack of a robust *in vitro* model of reversible latent infection in primary cells. This is partially explained by the observation that *in vitro* infection of MDM is associated with continuous virus production for several weeks in culture, likely reflecting an absent or reduced virus-dependent cytopathicity^45,46^. However, Brown and colleagues have provided evidence of reversible latent HIV-1 infection in primary human MDM infected with an EGFP-coding virus by purifying EGFP negative cells containing integrated provirus that could be reactivated by cell stimulation with IL-4^47^. Additional evidence of a potential latent state of HIV-1 infection of primary human MDM was earlier associated with cell stimulation by IL-10, although this cytokine also interfered with a pre-integration step of the virus life cycle^48^, making difficult to dissect out the contribution of the inhibition of proviral transcription from the global inhibition of virus replication induced by IL-10 stimulation.

In this context, we have earlier reported that short-term (18 h) stimulation of primary human MDM with the pro-inflammatory cytokines IFN-γ and TNF-α, an experimental condition commonly referred to as “M1 polarization”^30^, before infection caused a significant containment of virus replication initially attributed to an impairment of viral entry^21^, consistently with earlier observations^49^. In a subsequent study^22^, we described a strong association between the HIV-restrictive profile of M1-MDM and the induction of expression of APOBEC3A that was previously described to act as a restriction factor for HIV-1 infection of monocytes freshly isolated from peripheral blood^29^. As this restrictive profile was induced by stimulation of MDM shortly before infection, we have here investigated the consequence of re-exposing infected M1-MDM to the same stimuli 7 days after infection, a time window sufficient to allow the establishment of a pool of cells harboring integrated proviruses. We initially hypothesized that restimulation of infected M1-MDM with pro-inflammatory cytokines known to be potent upregulators of proviral transcription^23–26^ would lead to a burst of virus production from these cells. In sharp contrast, we consistently observed a stronger containment of virus replication, frequently approaching the limit of detection of our RT activity assay, in comparison to infected M1-MDM that were not restimulated. As we excluded that this quasi-silent phenotype could be consequent to either cytokine-dependent cytotoxicity^50^ or accumulation of preformed virions in VCC^27^, we switched our hypothesis to consider M1 restimulation as a mean to drive primary human macrophages towards a state of proviral latency. Indeed, restimulation of infected M1-MDM with IFN-γ and TNF-α upregulated the expression of restriction factors APOBEC3A and APOBEC3G that could likely account for the decreased levels of HIV DNA observed in both M1-MDM and M1^2^ MDM^2,3^. Of interest, SAMHD1, a hydroxylase inhibited by the HIV-2 associated protein Vpx, which acts by reducing the availability of the pool of deoxynucleotides required for the RT step preceding proviral integration^28,51^, was not significantly modified in terms of its expression in the different experimental conditions here explored. In this regard, a recent study showed that a significant fraction (up to 20%) of both microglia and monocyte-derived tissue resident macrophages, unlike MDM, is in a G1-like state of their life cycle that, although not coupled with cell proliferation, allowed these cells to bypass the restriction imposed by SAMHD1^52^.

Restimulation of M1-MDM with M1 cytokines 8 days after infection failed to upregulate both US and MS viral transcripts that remained barely detectable also at later time points. This partially unexpected finding was not explained by the lack of STAT1 and NF-kB activation, two transcription factors previously linked to activation of proviral transcription both in T lymphocytes and myeloid cells^23–26^. We hypothesize that their inductive effects on proviral transcription was overcome by the simultaneous expression of TRIM22 and CIITA, two inducible restriction factors acting as negative transcriptional regulators of HIV expression. In this regard, we have earlier shown that TRIM22 interferes with Sp1 driven proviral transcription^31,32^ whereas other investigators have reported its negative effects also in MDM^53,54^. CIITA, in addition to its key role of the upregulation of MHC Class II antigens^55^, was demonstrated to compete with Tat for binding to the P-TEFb complex^56^, also in myeloid cells^33^, thereby resulting in the downregulation of HIV-1 gene expression. More recently, we have reported that TRIM22 and CIITA can aggregate in nuclear bodies also containing TRIM19/PML and Cyclin T1 (a component of the P-TEFb complex)^34^ suggesting that these factors may act in concert.

However, we suggest that STAT1 and NF-kB activation could still play a role in terms of “priming” of the selective upregulation of virus production observed in M1^2^ MDM, but not in control or M1-MDM, cocultured with PHA blasts or incubated with their filtered supernatant. In this regard, the observation that Blast Sup induced virus production from M1^2^ MDM indicated that, on the one hand, cell-to-cell contact between infected MDM and PHA blasts was dispensable and, on the other hand, that virus replication occurred mostly, if not only, in M1^2^ MDM rather than in PHA blasts (that were maintained in medium devoid of IL-2 to prevent their proliferation). The observation that addition of lamivudine/3TC completely prevented the inductive effect of PHA blasts or of their supernatant on virus production from M1^2^ MDM (as well as on the minimal levels of virus replication observed in unstimulated cells) demonstrates, on the one hand, that these cells harbored replication-competent virus and, on the other hand, that most of the released virus derived from newly infected cells rather than from persistently infected cells as observed in CTR and M1-MDM whose virus production was insensitive to the presence of the RT inhibitor. In this regard, in order to provide formal evidence that at least a fraction of M1^2^ MDM harbored latently infected proviruses we reproduced the same profiles of virus restriction and reactivation upon infection of CTR, M1- and M1^2^ MDM with VSV-G pseudotyped HIV-1 that undergoes a single round of infection and it is incapable of viral spreading.

Thus, M1^2^ MDM represent a robust model of low levels virus replication and reversible HIV-1 latency in primary human macrophages that could be further exploited for testing latency-reversing and/or latency-enhancing agents in these cells. Our study also emphasizes the potential role of restriction factors of the APOBEC family and of physiological negative regulators of proviral transcription, such as TRIM22 and CIITA, in infected primary human macrophages, at least *in vitro*. As, at least in the case of TRIM22, allelic variants affecting its coding sequences have been linked to differential outcomes of HIV-1 disease^57^, it is tempting to speculate that they could also differentially affect the nature and size of the inducible viral reservoir in infected individuals receiving cART.

## Methods

### Reagents

Human, endotoxin-free recombinant cytokines IFN-γ and TNF-α were purchased from R&D Systems (Minneapolis, MN, USA); Ficoll-Hypaque and Percoll density gradient were bought from Amersham Biosciences Europe (Milan, Italy) and from GE Healthcare (Fairfield, CT), respectively. Dulbecco’s modified Eagle’s medium (DMEM), phosphate-buffered saline (PBS), fetal bovine serum (FBS), normal human serum (NHS), penicillin, streptomycin (pen/strep) and glutamine were purchased from Lonza (Cologne, Germany). Polyadenylic Acid polyA and oligo d(T)_12–18_ were purchased from Amersham Pharmacia Biotech (Uppsala, Sweden); [α-^32^P]-dTTP was purchased from Perkin Elmer (Waltham, MA, USA); DE81 paper was purchased from Whatman (Maidstone, UK). SSC (0.3M sodium citrate in 3 M NaCl), KCl and phorbol-12, 3TC (Lamivudine) and Phytohemagglutinin (PHA) were purchased from Sigma-Aldrich (St. Louis, MO, USA); MgCl_2_ was purchased from BDH lab Supplies (Poole, U.K.); Accutase was purchased from Sigma Aldrich (Merck KGaA, Darmstadt, Germany).

### Isolation of human monocytes from peripheral blood mononuclear cells (PBMC) and differentiation into monocyte-derived macrophages (MDM)

PBMC were isolated from the buffy coats of healthy HIV-seronegative blood donors by Ficoll-Hypaque density gradient centrifugation. Monocytes were then purified by Percoll density gradient centrifugation reaching 80–90% purity, as determined by CD14 expression and other lineage markers, as described^46^. The cells were then washed, resuspended in DMEM containing pen/strep (1%), glutamine (1%), heat-inactivated FBS (10%) and NHS (5%) (Complete Medium). Monocytes were seeded into 48-wells plastic plates at the concentration of 5 × 10^5^ cells/mL and cultivated for 7 additional days at 37 °C in 5% CO_2_ to promote their full differentiation into MDM (≥95% CD14^+^), as previously described^21^.

### M1 and M1^2^ polarization of MDM

Fully differentiated MDM were stimulated for 18 h with IFN-γ (20 ng/ml) and TNF-α (2 ng/ml) to induce M1 polarization^21^. Then, the medium containing polarization cytokines was removed and replaced with complete medium before HIV-1 infection. In order to obtain M1^2^ MDM, infected M1-MDM were restimulated in the same conditions described above with IFN-γ (20 ng/ml) and TNF-α (2 ng/ml) 7 days after infection, as indicated in Fig. 1A. The cytokine containing medium was removed 18 h after restimulation and complete medium was added to the adherent cell cultures.

### CCR5-dependent (R5) HIV-1 infection of MDM

Both CTR and M1-MDM were infected with the macrophage-tropic, laboratory-adapted R5 HIV-1_BaL_ strain at the multiplicity of infection (m.o.i.) of 0.1. Multiple aliquots of culture supernatants were collected every 3 days up to 24 days post-infection and stored at −20 °C to assess virus production. At the end of each infection experiment, supernatants were thawed and analyzed for their viral content by measuring the levels of virion-associated Mg^2+^-dependent RT activity present in the supernatant, with a radioactive assay. As 99% of the RT enzyme is particle-associated, this assay is a faithful indication of the production of new progeny virions^58^.

### VSV-G enhanced green fluorescence protein (eGFP) pseudotyped HIV-1 infection

The HIV-1 vector and plasmid used in this study have been described previously^59^. HIV-eGFP virus (in which *nef* was replaced with the eGFP reporter gene) was produced by cotransfection with a ratio of 1:7 of pMD2.G together with pNL4–3_GFP_R-Env-^60^ (both plasmids were obtained from the NIH AIDS Research and Reference Program, Division of AIDS, NIAID, NIH, Bethesda, Maryland, USA). Vector containing supernatants were harvested 48 h after transfection in 293 T cells, cleared by centrifugation, filtered by a 0.45 mm filter (MILLEX-HV PVDF; Millipore, Carrigtwohill, County Cork, Ireland), and stored at −80 °C.

CTR, M1-MDM and M1^2^ MDM were infected with 60 µl/well of HIV-eGFP viral stock. Infected MDM (5 × 10^5^ cells/condition) were detached from plastic adhesion after incubation with Accutase (150 µl/well for 30 min at 37 °C), as described^46^, spun, and their pellet was resuspended in a fixing solution containing 4% paraformaldehyde (PFA). Flow cytometry for GFP expression was performed using a FACS Calibur instrument (Becton Dickinson Italia, SpA, Milano, Italy), and the results were analyzed with the FlowJo software version 8.4.3 (Tree Star, Ashland, Oregon, USA).

### Stimulation of infected MDM with allogeneic PHA blasts or their culture supernatant

Human PBMC of different donors were stimulated for 72 h with PHA (5 μg/ml) in order to induce their blast transformation. Cells were then washed and resuspended in RPMI 1640, 10% FBS without supplementation of IL-2 in order to prevent their cellular proliferation. Culture supernatants from PHA blasts were collected after 72 h and filtered through a 0.45 µm filter before incubation with MDM.

### Cytotoxicity assay

ToxiLight^TM^ bioassay kit (Lonza, Cologne, Germany) was used to check the potential citotoxicity at different times post-infection in the different experimental conditions. This kit quantitatively measures the release of adenylate kinase (AK) activity from damaged cells, providing an accurate and sensitive determination of cytolysis^61^.

### PCR-based quantification of HIV-1 *gag* DNA

MDM were washed, lysed and treated with proteinase k at 65 °C for 2 h and then at 95 °C for 15 min. An aliquot of cell lysate corresponding to 1 × 10^6^ cells was incubated in a PCR mix containing the following HIV-1 *gag* gene primer pair and probe: forward 5′-acatcaagcagccatgcaaat-3′; reverse 5′-atctggcctggtgcaatagg-3′; probe 5′-(FAM)catcaatgaggaagctgcagaatgggataga(TAMRA)-3′. The number of HIV-1 DNA copies was then determined by interpolating a reference standard curve (showing a linear distribution between 10^1^ and 10^7^ copies, r = 0.99) after normalization for human glyceraldehyde 3-phosphate dehydrogenase (GAPDH) DNA copy number (forward primer 5′-accacagtccatgccatcact-3′, reverse primer 5′-ggccatcacgccacagtt-3′ and probe, 5′-(FAM)cccagaagactgtggatggcccc(TAMRA)-3′). The thermal cycling conditions were 50 °C for 2 min, 95 °C for 12 min, and 40 cycles of 95 °C for 15 s and 65 °C for 1 min^62^.

### Quantification of integrated (proviral) DNA by Alu-PCR

The levels of integrated proviral DNA were estimated adopting the published Alu-PCR protocol, involving a nested PCR-based assay with two sets of Alu-LTR primers and a probe, with minor modifications^63^. Genomic DNA (100 ng) was first amplified with AccuPrime Taq DNA Polymerase High Fidelity (Life Technologies Italia, Monza, Italy) in a DNA thermal cycler (Perkin Elmer, Waltham, Massachusetts, USA). Then, a real-time PCR was performed with an aliquot equivalent to 1/10th of the 25th cycle PCR product with the second-round LTR primers and TaqMan probe. For control, all samples were also amplified with a primer pair and probe targeting mitochondrial DNA. Standard curves for both target and normalizer were obtained by nested PCR, as described above, using serially diluted genomic DNA (from 500 to 0.16 ng). The levels of proviral DNA, calculated from the standard curves, were expressed as ng of integrated provirus normalized to those of a mitochondrial DNA standard curve.

### Quantification of HIV-1 RNA by real-time PCR

HIV-1 RNA, both unspliced (US) and multiply spliced (MS), was analyzed 8 days after infection. Total RNA was extracted by using TRIzol reagent and PureLink RNA Mini Kit (Ambion, Life Technologies) and cDNA was synthesized from 1 µg total RNA. Multiplex TaqMan qPCR (Applied Biosystems) was performed with 50 ng of cDNA in a total volume of 25 µl with the following primer pairs and probe: forward 5′-cagactcatcaagcttctctatcaaa-3′, reverse 5′-tcactaatcgaatggatctgtctc-3′, probe RNAS 5′-(FAM)acccgacaggcccgaaggaa(TAMRA)-3′ (for MS RNA); forward 5′-acatcaagcagccatgcaaat-3′, reverse 5′-atctggcctggtgcaatagg-3′; RNA gag probe 5′-(FAM)catcaatgaggaagctgcagaatgggataga(TAMRA)-3′ (for US RNA). mRNA expression was calculated by using the relative quantification method (∆∆Ct) to uninfected CTR MDM all normalized to human 18S ribosomal RNA expression using Taqman ribosomal RNA control reagent kit (Applied Biosystems). All reactions were performed with an ABI 7700 Prism instrument (Applied Biosystems) using the following thermal cycling conditions: 50 °C for 2 min, 95 °C for 15 min, and 40 cycles of 95 °C for 15 sec and 60 °C for 1 min.

### Quantification of cellular transcripts by real-time PCR

Relative expression of TRIM22, CIITA, CdK9 and Cyclin T1 RNA was analyzed at day 0 before infection and 8 days post-infection. Total RNA was extracted from cells using TRIzol reagent. cDNA was synthesized from 0.5 μg total RNA; cDNA (25 ng for each reaction) was amplified by PCR by using an ABI Prism 7000 Sequence Detection System (Applied Biosystems) with IQSYBR Green PCR master mix (Bio-Rad) according to the manufacture’s protocol. All mRNA values were normalized to Ribosomal Protein S7 (RPS7) mRNA. For each gene analyzed the mRNA level in CTR MDM at day 0 was set to 1. The following primer pair sets were used: CIITA, forward 5′-ggatcctcacggcctttt-3′, reverse 5′-ccccgatcttgttctcactc-3′; TRIM22, forward 5′-ggcttggtgagtgaatctgg-3′, reverse 5′-tcacaaactcctgcagtgct-3′; CdK9, forward 5′-ttcggggaggtgttcaag-3′, reverse 5′-atctcccgcaaggctgtaat-3′; CyT1, forward 5′-ggcgtggacccagataaag-3′, reverse 5′-ctgtgtgaaggactgaatcat-3′; RPS7, forward 5′-tggagatgaactcggacctc-3′, reverse 5′-cgaccaccaccaacttcaa-3′.

### APOBEC3A/G, SAMHD1, NF-kB and STAT1 detection by Western blotting

MDM were lysed in NP40 buffer [50 mM Tris HCl (pH 7.5), 150 mM NaCl, 1% NP40 and 0.5% w/v deoxycholate] containing protease and phosphatase inhibitors. Proteins were separated by 10% (for STAT1 and NF-kB detection) or 7,5% (for APOBEC3A/G and SAMHD1 detection) SDS-PAGE, transferred to a nitrocellulose membrane by electroblot analysis and probed with the following antibodies (Ab): rabbit anti-APOBEC3A/G polyclonal Ab, kindly provided by Dr. Klaus Strebel (Laboratory od Molecular Microbiology, National Institute of Allergy and Infectious disease, National Institute of Health, Bethesda, MD, USA); polyclonal rabbit anti-STAT1 and monoclonal anti-phospho-STAT1 (tyr701) Ab (mAb; Cell Signaling Technology Inc., Denvers, MA, USA); mouse anti-NF-kB p65 and anti-phospho-p65 (ser536) mAbs (Termofisher Scientific, San Diego, California, USA; polyclonal rabbit anti-SAMHD1 Ab (Sigma-Aldrich, St. Louis, MO, USA); rabbit anti-GAPDH mAb was used as control (14C10 clone; Cell Signaling Technology, Inc., Denvers, MA, USA).

### Statistical analysis

Statistical analysis was performed using the Prism GraphPad software v. 6.0 (GraphPad Software, www.graphpad.com). Results are reported as means ± SD. Comparison between groups was performed using the parametric one-way ANOVA; p values < 0.05 were considered significant. To control for inter-donor variability, all assays were performed in triplicate samples per condition with MDM derived from independent donors. T-test analysis was performed when appropriate, as indicated in the text.

### Ethical Approval

The use of human peripheral blood cells derived from buffy coats for experimentation has been approved by the San Raffaele Hospital Ethical Committee; volunteers blood donors (>18 years old) sign a statement that material not of medical use could be utilized for research, noncommercial purposes. All methods were carried out in accordance with relevant guidelines and regulations approved by the aforementioned Institutional Ethical Committee; experiments involving infectious material (HIV) were conducted in BSL-3 laboratories.

## Electronic supplementary material

Supplementary Material

## Data Availability

The datasets generated during and/or analyzed during the current study are available from the corresponding author on reasonable request.
