# Supplementary material for: Reversible Human Immunodeficiency Virus Type-1 Latency in Primary Human Monocyte-Derived Macrophages Induced by Sustained M1 Polarization
Source: Sci Rep. 2018 Sep 24;8:14249. doi: 10.1038/s41598-018-32451-w (PMC6155284; doi:10.1038/s41598-018-32451-w)
Supplement: Supplementary file 1 — Supplementary Material [file 41598_2018_32451_MOESM1_ESM.docx]

**Supplementary Material**

**Reversible Human Immunodeficiency Virus Type-1 Latency in Primary Human Monocyte-Derived Macrophages Induced by Sustained M1 Polarization**

Francesca Graziano ^1^, Giulia Aimola^1^, Greta Forlani^2^, Filippo Turrini^1^,

Roberto S. Accolla^2^, Elisa Vicenzi^1^ & Guido Poli^1,3^*

^1^Division of Immunology, Transplantation and Infectious Diseases, San Raffaele Scientific Institute, Milano, Italy; ^2^Department of Medicine and Surgery, University of Insubria, Varese, Italy; ^3^Vita-Salute San Raffaele University School of Medicine, Milano, Italy.

##
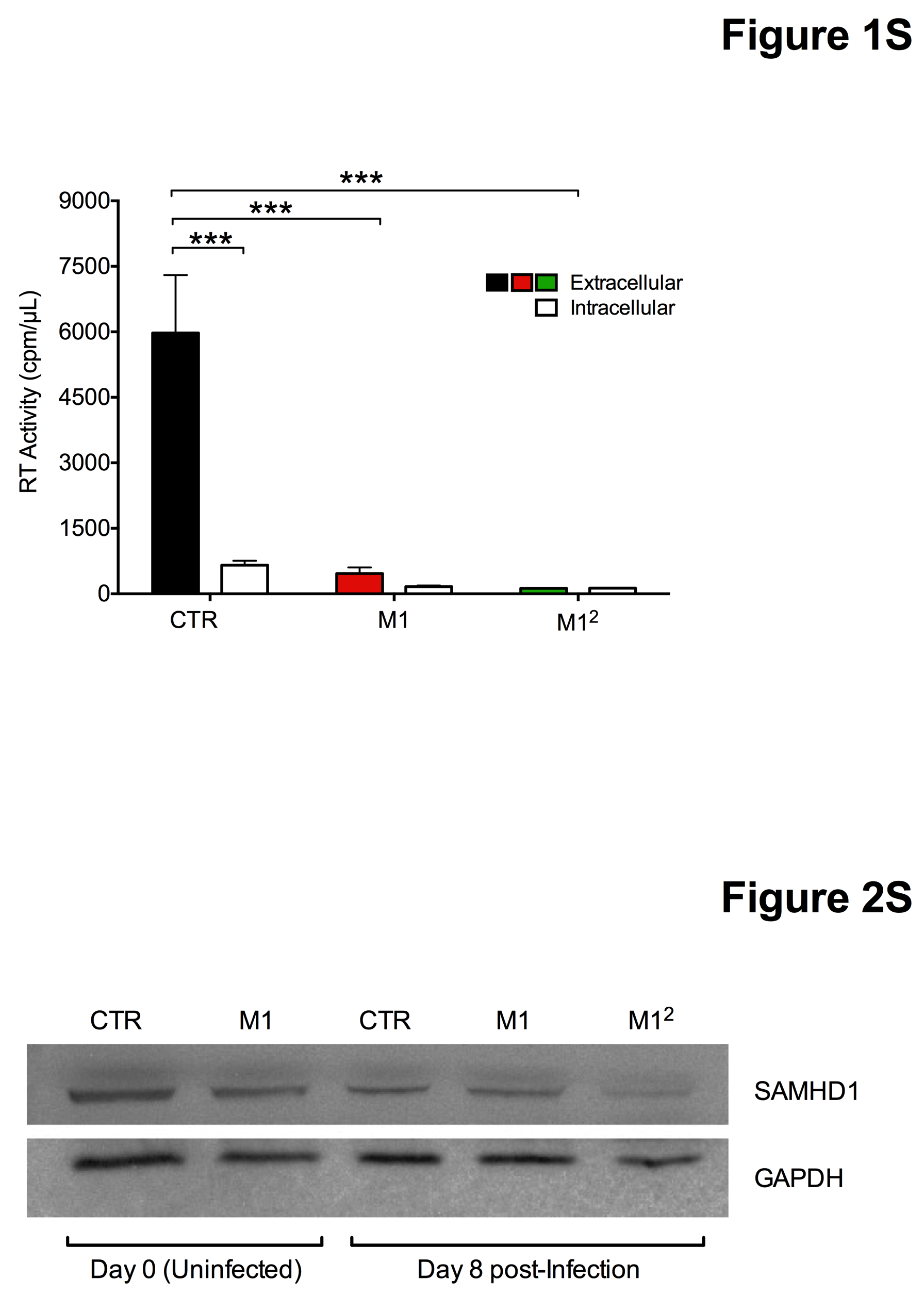


## Figure S1. Lack of cell-associated RT activity in M1-MDM and M1^2^ MDM. To rule out the possibility that the significantly reduced levels of RT activity released in the culture supernatants of M1^2^ MDM was the consequence of virion accumulation in VCC or particle adhesion to the cell surface 12-days infected cells were incubated with medium containing 50 μl of NP-40 lysis solution (50 mM Tris-Cl pH: 8.0, 150 mM NaCl, 1% Triton X-100) in order to disrupt the cellular membrane. Cells were then kept in agitation for 15 min to facilitate the lysis and were then resuspended in 450 μl of complete medium before measuring the levels of RT activity. However, no evidence of virion accumulation or adsorption were obtained in these experimental conditions. Statistical analysis by On-way ANOVA; ***p <0.001


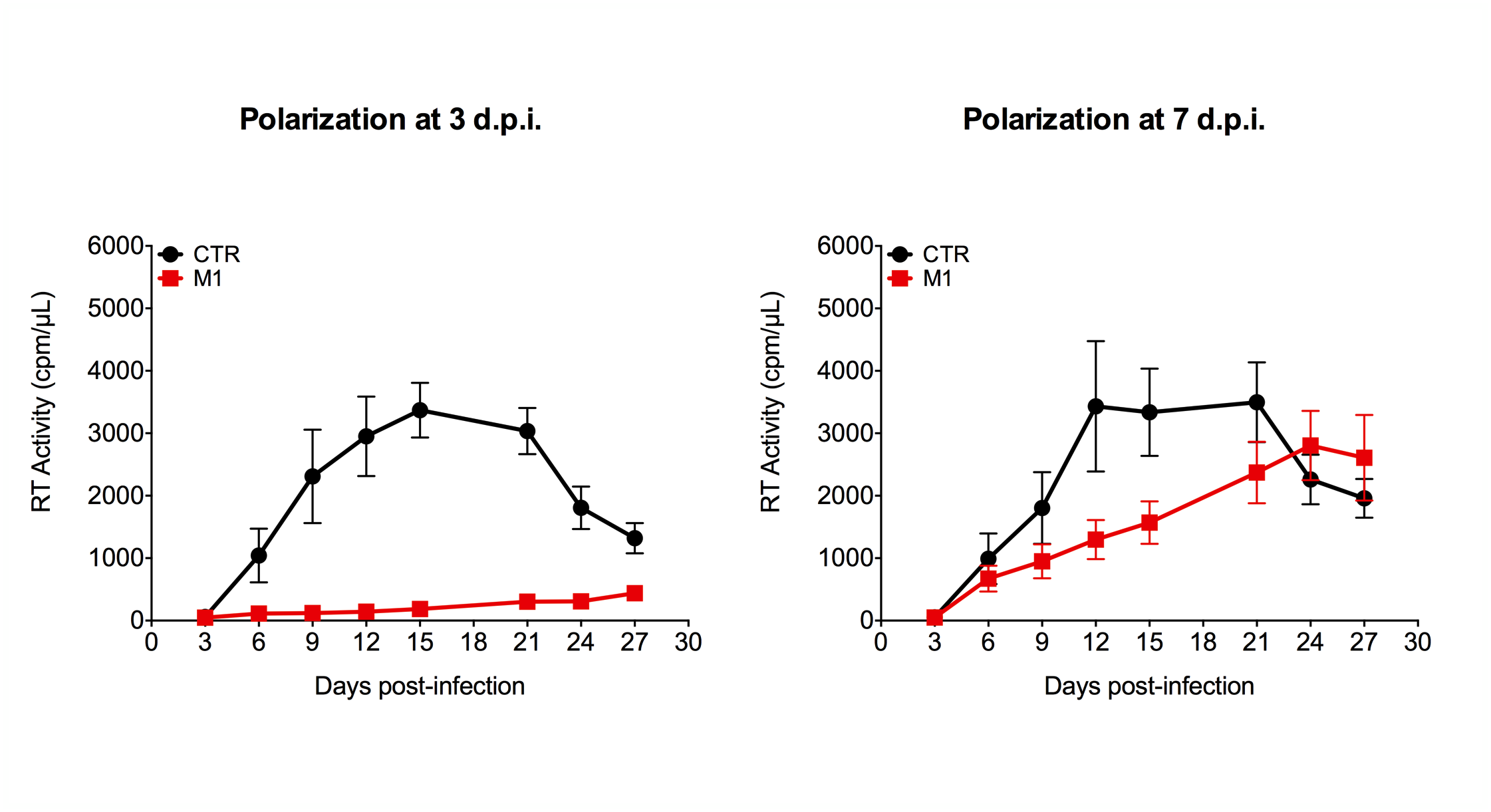


(n=4)

**Figure S2. Moderate inhibition of HIV-1 replication in MDM by M1-polarization induced 7 days after infection.** MDM were differentiated and infected as previously discussed. Seven days after infection cells were stimulated or not with IFN-γ and TNF-α (M1 polarization) resulting in moderate levels of inhibition of virus replication, however much lower than those observed in M1^2^ MDM (as shown in **Figure 1B** and **C**). The graph illustrates the mean + SD of the infection of cells obtained from 4 independent donors.


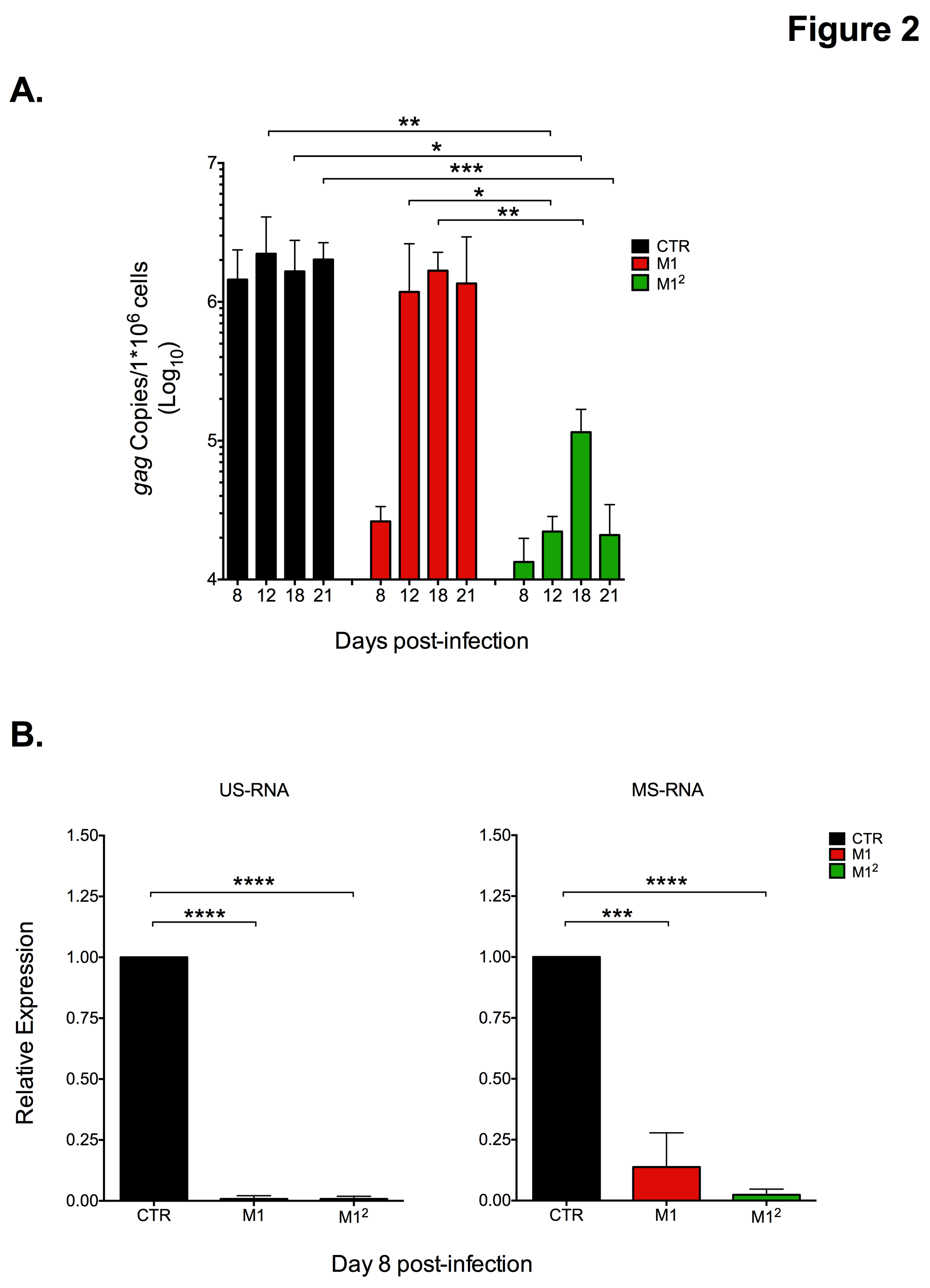


**Figure S3. M1^2^ MDM show a superior containment of HIV-1 *gag* DNA synthesis than M1-MDM.** HIV-1 *gag* DNA levels were lower than those of CTR infected MDM in both M1-MDM and M1^2^ MDM when analyzed 8 days after infection; however, at later time points only M1^2^ MDM maintained significantly lower levels of viral DNA vs. CTR and M1-MDM. Bar graphs represent the mean ± SD of the HIV-1 DNA copies obtained from the independent infections of cells isolated from 2 (day 8 post-infection), 8 (day 12 post-infection) and 4 (18 and 21 days post-infection) independent donors. P values were calculated by an unpaired t-test. *p<0.05, ** p <0.01, ***p <0.001.

**Figure S4. M1^2^ MDM show a repressed profile of HIV-1 RNA synthesis in the presence or absence of lamivudine/3TC.** The levels of expression of both US and MS HIV-1 RNA were verified at 8 and 21 days post-infection. At this latter time point part of the cell cultures were incubated with lamivudine/3TC (1 µM; n=2).


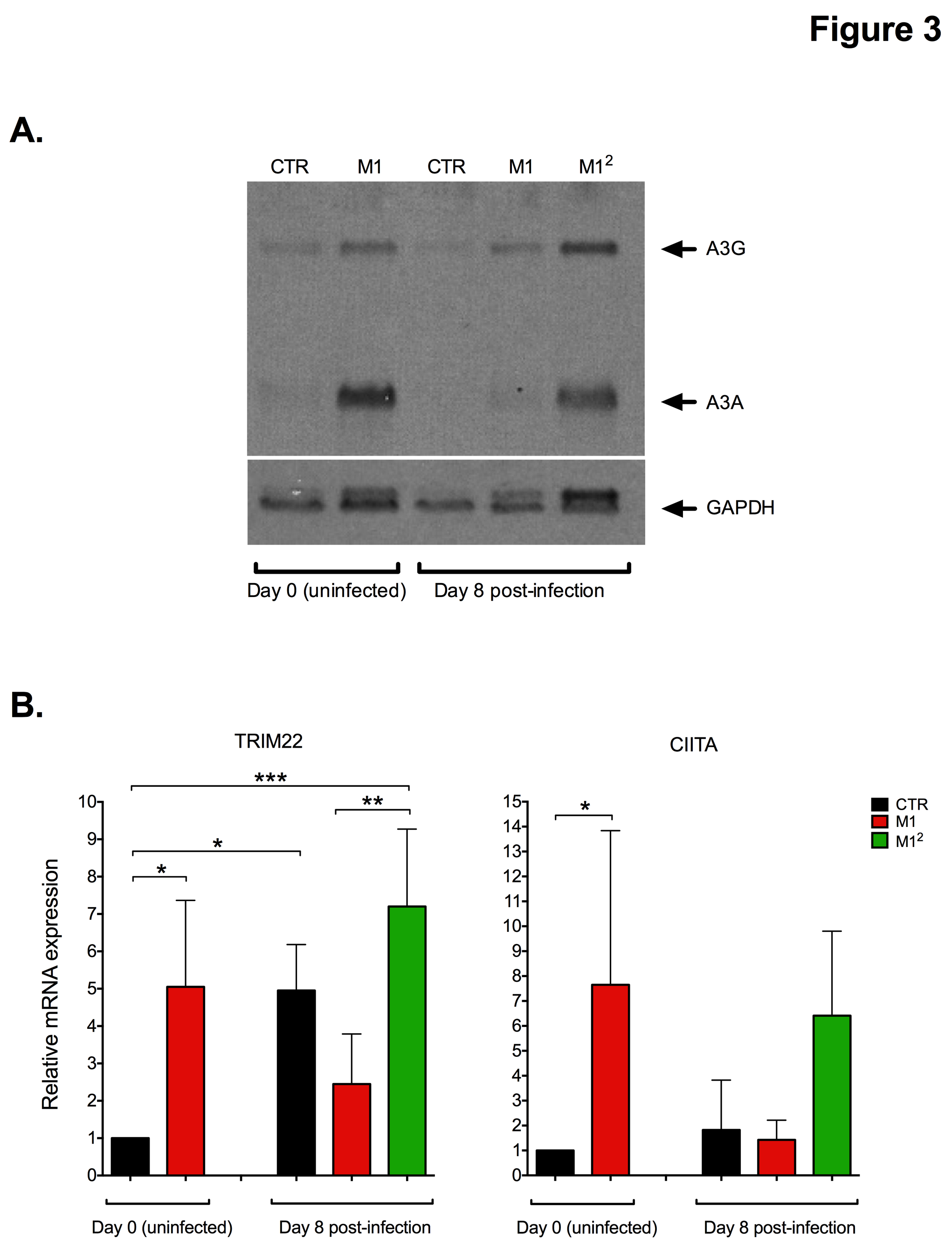

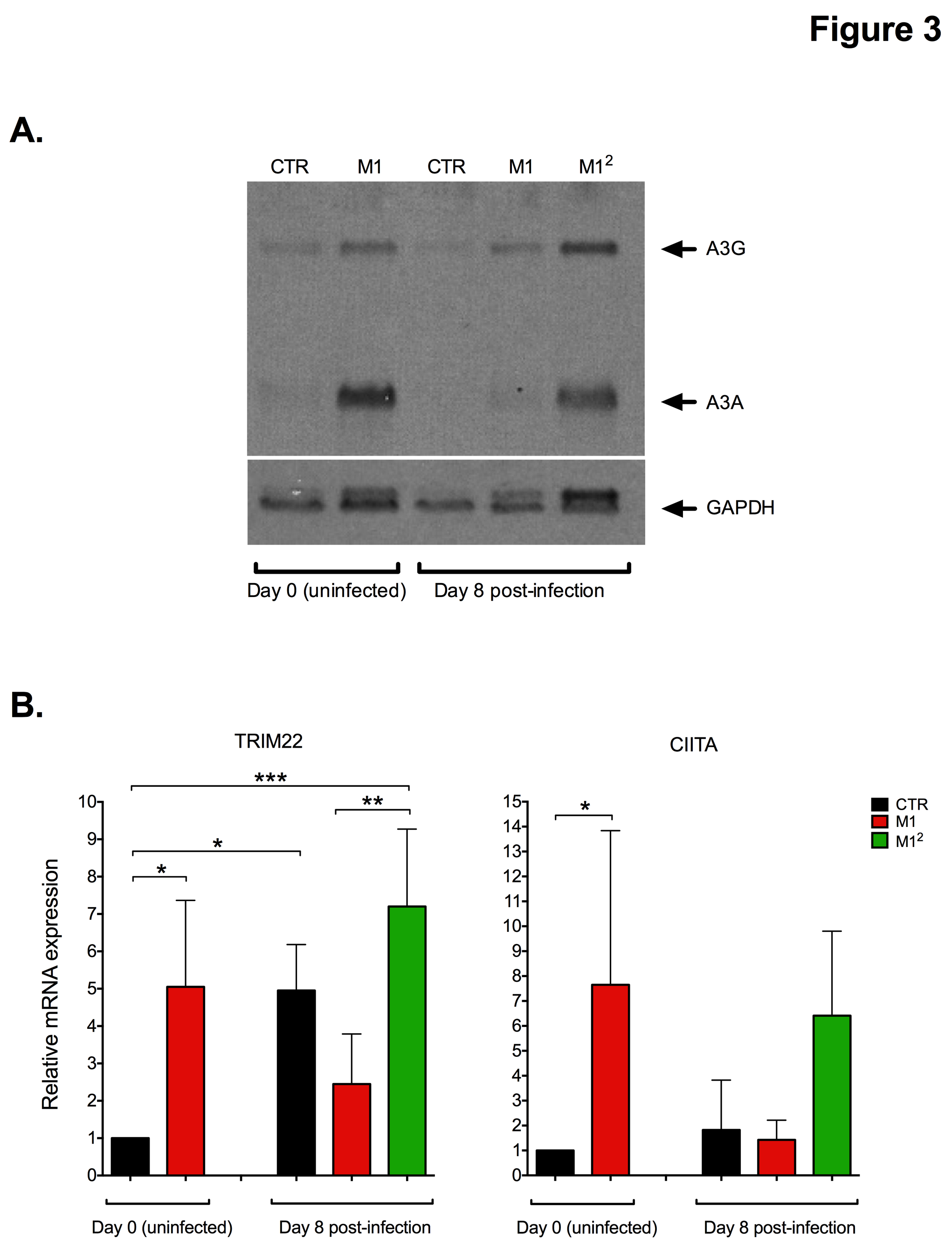


**Figure S5. Upregulation of HIV-1 restriction factors in M1-MDM and M1^2^ MDM.** Expression of APOBEC3A (A3A) and APOBEC3G (A3G). Cytokine stimulation induced the expression of A3A and upregulated that of A3G both before (Day 0) and 8 days after infection, particularly in M1^2^ MDM.


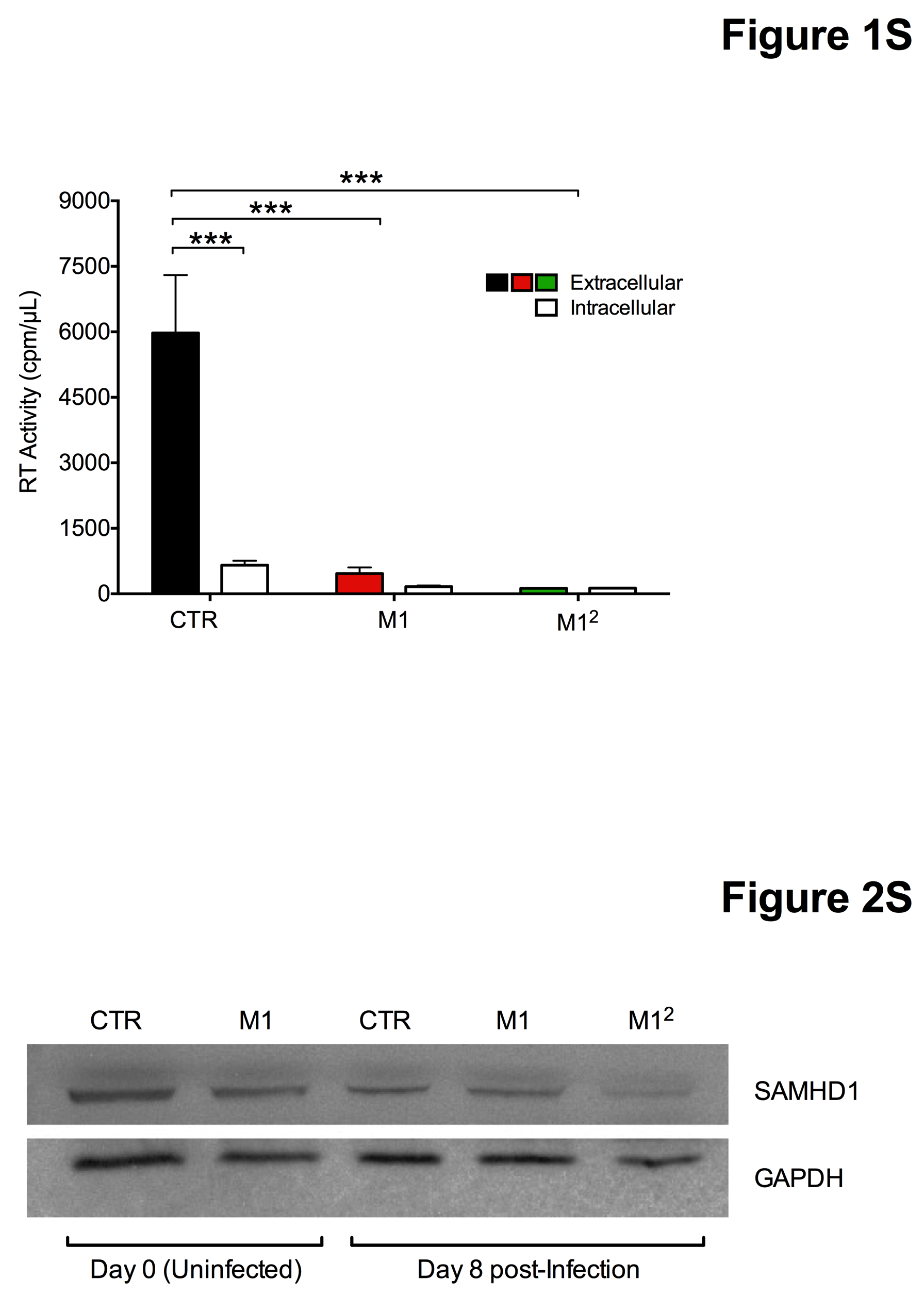


**Figure S6. Lack of modulation of the constitutive levels of SAMHD1 expression in CTR, M1-MDM or M1^2^ MDM.** Unlike what observed for APOBEC3A and 3G, Western blot analysis did not indicate significant changes in the levels of expression of SAMHD1 in the different experimental conditions tested.

**
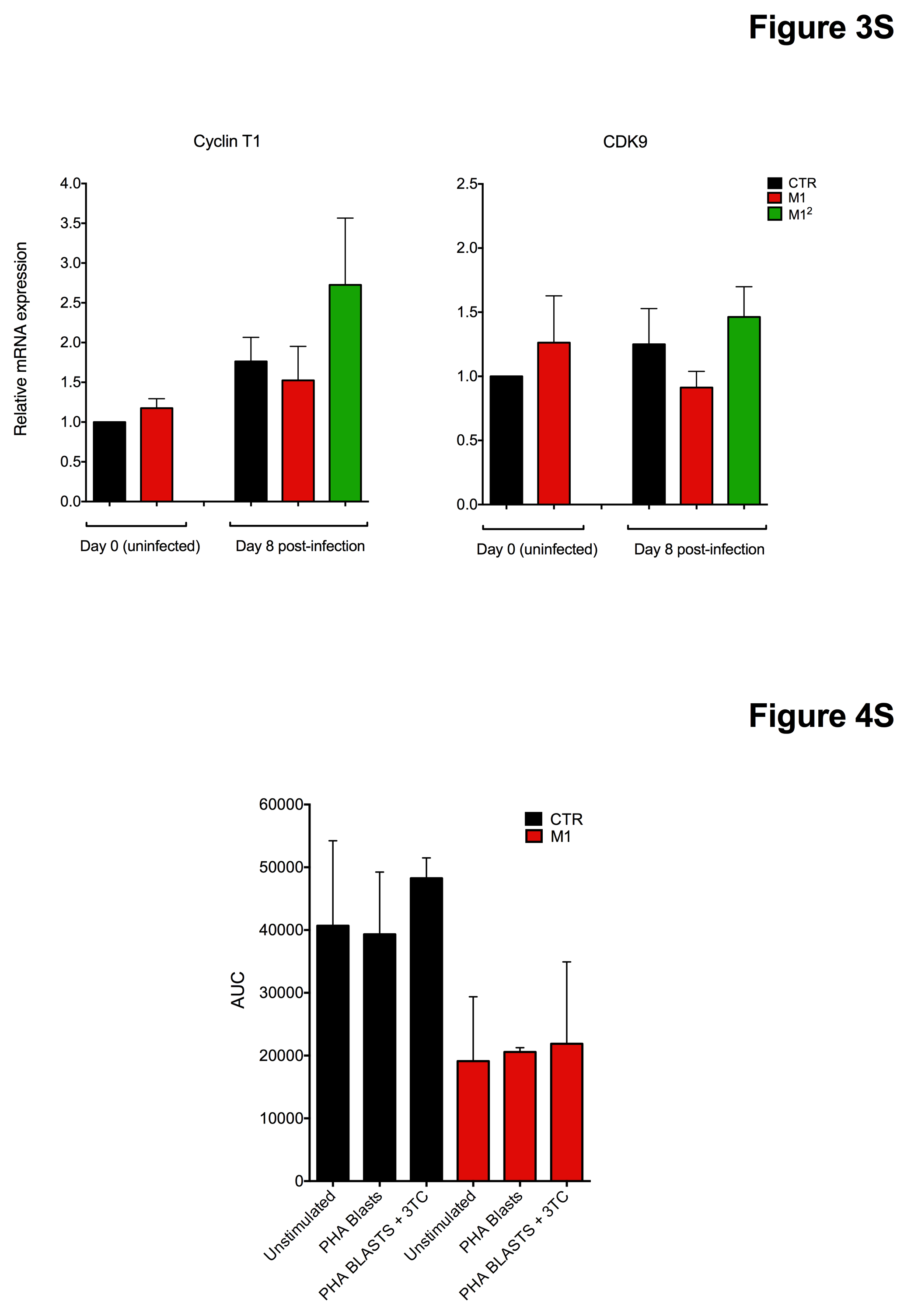
**

**Figure S7. Lack of modulation of PTEF-b by cytokine stimulation of MDM before and after infection.** Unlike what observed with TRIM22 and CIITA (see **Figure 3B**), the M1-polarizing cytokines failed to modulate the levels of cyclin T1 and CDK9 expression before and after infection.

**M1^2^**

**M1**

**CTR**


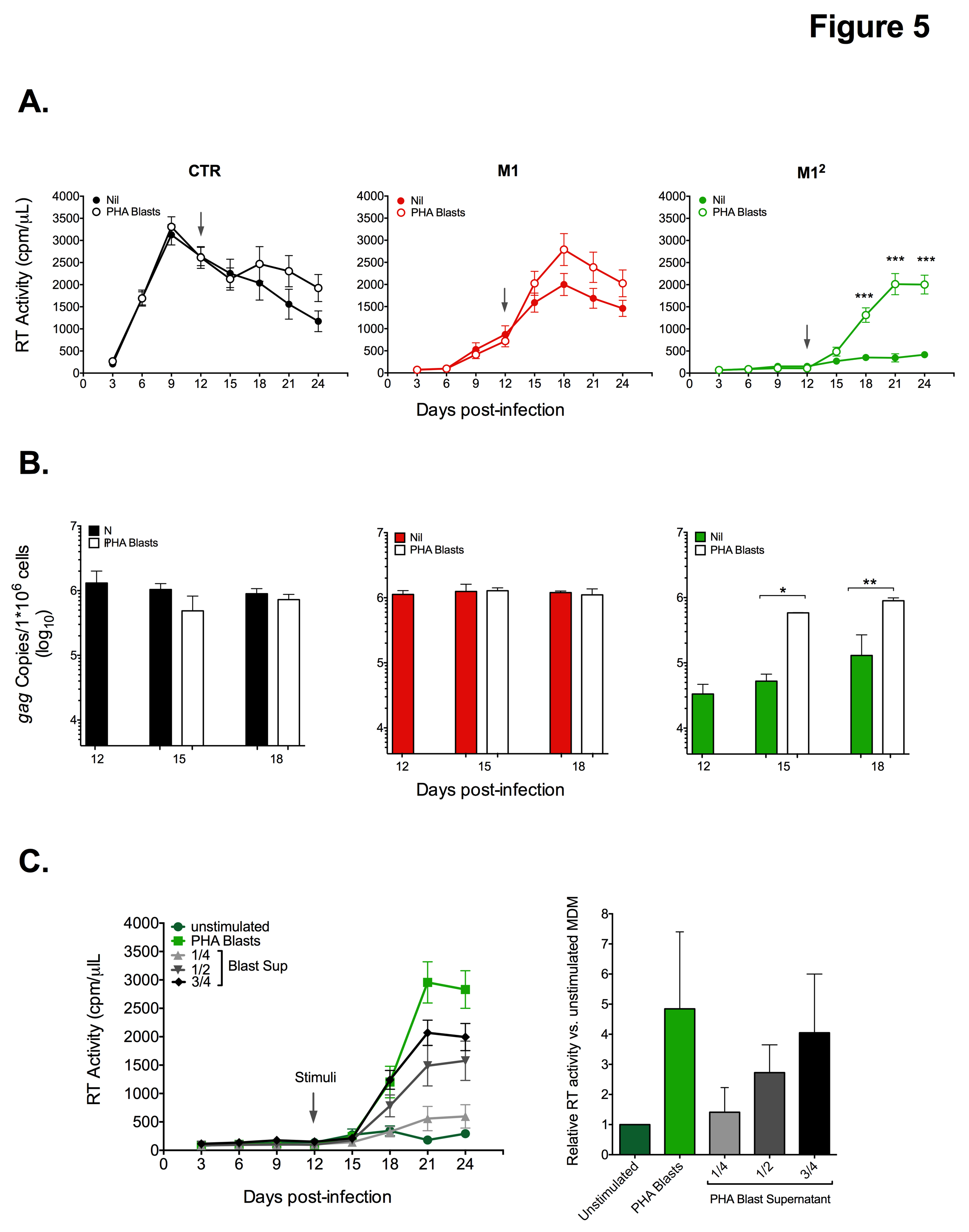


**Figure S8. Kinetics and levels of HIV-1 *gag* DNA in MDM in the presence or absence of PHA Blasts.** Bar graphs represent the mean ± SD of the HIV-1 DNA copies obtained from the infection of cells isolated from 2 independent donors. P values were calculated by unpaired t-test, *p <0.05, **p <0.01.

**
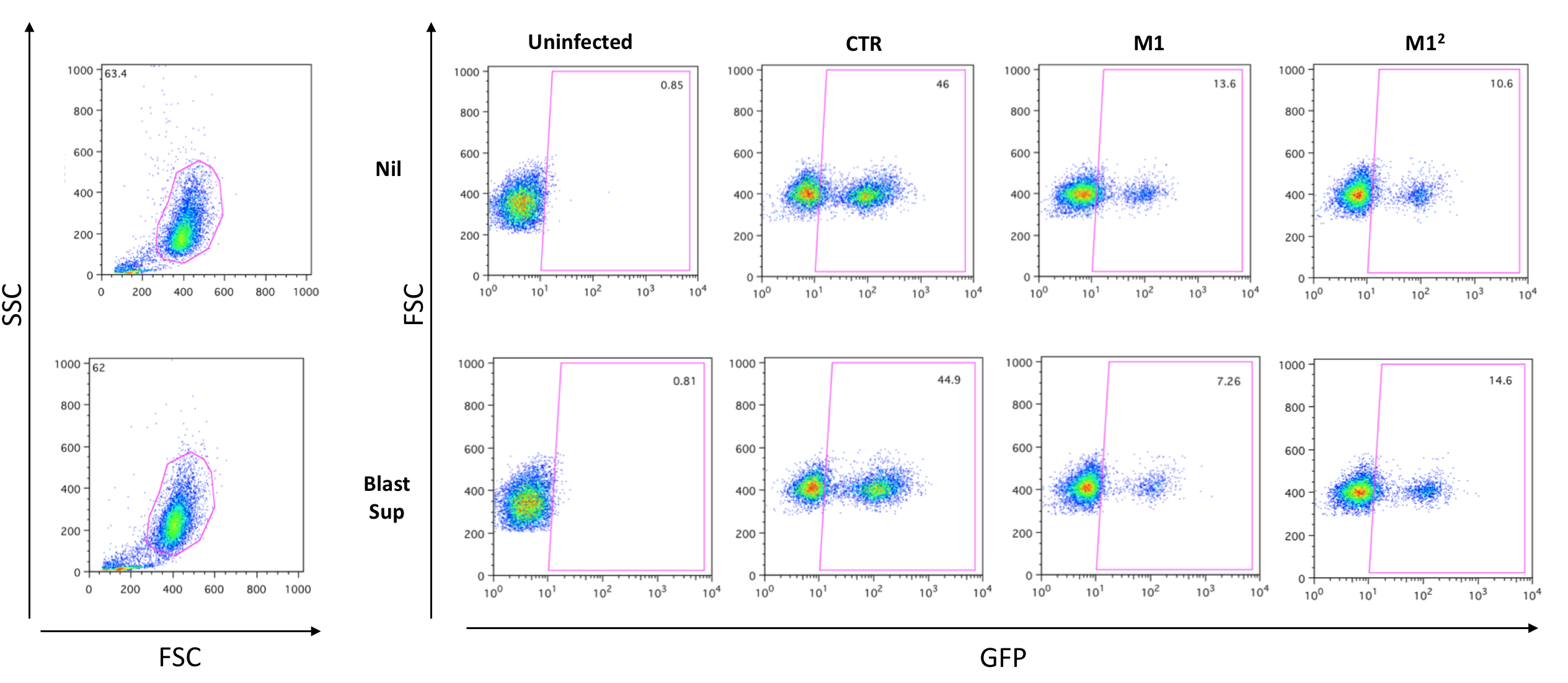
**

**Figure S9. eGFP expression after infection and stimulation of MDM with VSV-G pseudotyped virus.** CTR, M1-MDM and M1^2^ MDM obtained from cells of a single donor were infected with HIV-eGFP (60 µl of viral stock solution). After 13 days of culture and additional 72 h of stimulation with culture medium or Blast Supernatant, MDM (5x10^5^ cells/condition) were detached by Accutase, spun, and their pellet was resuspended in a fixing solution containing 4% paraformaldehyde (PFA). Flow cytometry for GFP expression was performed using a FACS Calibur instrument (Becton-Dickinson), and the results were analyzed with the FlowJo software version 8.4.3.
